# Prevalence of bereavement among current smokers in a state-wide cross-sectional surveillance survey

**DOI:** 10.18332/tid/208003

**Published:** 2025-09-11

**Authors:** Changle Li, Toni Miles

**Affiliations:** 1 School of Health Management, Fujian Medical University, Fuzhou, China; 2 College of Public Health, University of Georgia, Athens, United States; 3 Rosalynn Carter Institute for Caregivers, Americus, United States

**Keywords:** mental health, public health, smoking, grief, population survey

## Abstract

**INTRODUCTION:**

Research consistently shows that bereavement is associated with subsequent poor self-rated health. In a separate line of research, smoking is common among persons with a mental illness diagnosis. In a population-based survey, the following three hypotheses are tested: 1) Compared to non-smokers, smokers are not more likely to report bereavement; 2) Among the bereaved, demographic factors – gender, race, and age – do not influence the likelihood of being a current smoker; and 3) Smoking does not influence or mediate the effect of bereavement on poor self-rated health.

**METHODS:**

The sample consisted of 7354 respondents to the annual 2019 Georgia Behavioral Risk Factor Surveillance Survey (BRFSS). Multiple imputation, descriptive analysis, ordered logistic regression, and mediation models were used.

**RESULTS:**

With imputed datasets, we found that bereavement rates were higher among every day (52.2%) compared to former smokers (46.4%) and never smokers (43.3%). Bereaved persons who smoke are also more likely to report heavy drinking: females (OR=3.92; 95% CI: 2.96–5.18) and males (OR=3.64; 95% CI: 2.72–4.86). Bereavement rates are highest among males who report smoking some days (OR=52.7; 95% CI: 44.4–61.0) and among females who report smoking every day (OR=56.77; 95% CI: 50.9–62.7).

**CONCLUSIONS:**

Among all current smokers, bereavement is highly prevalent. However, gender, smoking and grief have a complex association. Bereaved female smokers typically smoke every day while bereaved male smokers on some days. Any bereaved smoker may benefit from cessation treatment to reduce health decline after loss.

## INTRODUCTION

Smoking carries significant risks for poor self-rated health, morbidity, and mortality^1,2^. Bereavement also carries significant risk for poor self-rated health, morbidity, and mortality^3-6^. Population surveys conducted worldwide consistently measure smoking prevalence. These same surveys do not measure bereavement. Heavy smoking is associated with dependence, and cessation leads to withdrawal symptoms such as anxiety, depression, insomnia, and difficulty concentrating^7^. These symptoms are also observed among the bereaved^8^. Collectively, these symptoms indicate emotional dysregulation and are predictive of cessation treatment failure with interventions such as bupropion or varenicline combined with cognitive behavioral therapy^9-11^. Functional MRI shows brain changes during grief similar to those observed among people with major depression or post-traumatic stress disorder, providing a biologically plausible rationale for a joint examination of smoking and bereavement^8^. There is consensus that bereavement is a risk factor for the development of Prolonged Grief Disorder (PGD, DSM F43.81) and Complicated Grief (CG, DSM F43.8)^12^. Seven to ten percent of bereaved persons will progress to PGD and/or CG^13^. Spontaneous recovery happens with 66.4% of complicated grief cases by the one-year mark^14,15^. The interaction of bereavement with smoking cessation represents a gap in our understanding of tobacco addiction and factors influencing successful cessation. Research is needed because the health and well-being of a broad circle of persons can be diminished by a single death^16-19^. The risk for poor self-rated health increases with the experience of 3 or more deaths in a 24-month span^20^. In one survey, 31.5% of adults who binge drink also report three or more losses in a 24-month span^21^. In the 2019 Behavioral Risk Factor Surveillance Survey, the US state of Georgia field tested a new bereavement module in its 2019 BRFSS. Based on this survey, 3673808 adults aged ≥18 years reported the death of family and/or friends within a 24-month window prior to the survey^20^.

In a national surveillance survey, there is evidence that participants are willing to discuss the deaths of family and friends. The Hungarostudy Epidemiological Panel Survey (n=4457) began with a cross-sectional survey of a nationally representative sample to study alcohol use in the three years after bereavement^22^. The Health and Retirement Survey (HRS) is a longitudinal, complex sampling survey of US adults aged ≥50 years, in which cohort members are recontacted every two years. The HRS item on bereavement was introduced in 2006, with response rates of ≥80%^23^. Analyses of HRS data have been used to identify individual mediators and moderators of health related to bereavement^5,17,24^. The HRS bereavement items were used for the 2019 Georgia BRFSS. Response rates to the individual items were ≥75%. In tests for response bias across four demographic subcategories – gender, age, self-reported race, and rural versus urban residence – no statistical differences were obtained^20^.

Self-rated health (SRH) measures the perceived well-being^25^. The item asks the respondent to assess their SRH in the 30 days prior to interview. SRH is utilized in population-level surveillance surveys worldwide. Poor SRH is consistently associated with smoking^26^. The findings of prevalent poor SRH in surveys as well as patient registries have led to the development of longer and more detailed clinical tools like Patient-Reported Outcomes Measures (PROMs) and the OECD study of persons living with chronic conditions primary survey (PaRIS)^26,27^. Cross-sectional data are an effective starting place when the goal is to account for the size of the population affected by poor SRH and to measure its association with smoking and bereavement.

Modeling of outcomes related to smoking or bereavement requires attention to the complex covariates associated with each. Each outcome is also subject to confounding, i.e. the influence of a 3rd variable that is not being studied, though not in the same way. Mediation modeling is an approach that parses direct and indirect effects of bereavement and smoking on an outcome common to both^28,29^. With this model, the size of the influence by a proposed mediator of the relationship between an independent variable and a dependent variable can be quantified. Mediation modeling can be applied to the relationship between bereavement and SRH. The total effect of bereavement on SRH can be parsed between a direct effect of bereavement and an indirect effect of smoking. Population surveillance surveys that measure smoking, bereavement, and SRH are an ideal resource for developing hypotheses derived from mediation modeling.

In previous analyses, two mediation models were used to measure the influence of bereavement on the four domains of health – SRH, Physical Health, Mental Health, and Activity Limitation^4^. A separate model for indirect effects – one for obesity and the other for smoking – on each domain was done. Obesity did not have a significant indirect effect on any of the domains. Smoking had significant coefficients ranging from 0.72 to 1.10, with the most considerable total effect observed for poor SRH (0.66; 95% CI: 0.41–0.90). In the smoking model, the ratio of the indirect effect to the total was 48.48%. One interpretation of these analyses is that both bereavement and smoking may equally influence poor SRH. In sum, the current analysis examines the association between current smoking and recent bereavement to test the following three hypotheses:

1. Current smokers are not more likely to report bereavement in the prior 24 months compared to persons who have never smoked.
2. Other covariates such as gender, race, and age are not more likely to influence the likelihood of smoking among the bereaved.
3. Smoking does not influence or mediate the effect of bereavement on poor SRH.

## METHODS

### Study design

This is a secondary dataset analysis of the Behavioral Risk Factor Surveillance System (BRFSS), an annual observational, cross-sectional surveillance survey conducted in all 50 US states^30^. Its data are used to design and test public health interventions. BRFSS queries major chronic health conditions, health-related risk behaviors, and the use of preventive services. In this analysis, the data are limited to the state of Georgia. Georgia is the only state to add a new bereavement module to its 2019 field survey. The data used in this analysis can be obtained from the US Georgia Department of Public Health (https://dph.georgia.gov/phip-data-request).

### Setting and participants

The 2019 BRFSS includes 7354 adults aged ≥18 years. To overcome randomly distributed missing data, multiple imputation techniques were applied to increase the precision of estimates and reduce bias. Supplementary file Table S1 shows missing rates for items included in the analyses. Smoking status was available for 6847 persons (93.1%), with 507 (6.9 %) missing a response to this item. Due to the differential loss of responses to both individual core and state-added modules, 917 individuals are missing from the group that responded to the bereavement item in the 2019 BRFSS. By design, BRFSS can be used in two ways. It can be used as a panel survey (without sampling weights) or as a resource that utilizes its full complement of sampling and weighting features. Based on an analytic sample of 7354 adults aged ≥18 years, the combination of sampling weights and MI techniques is used to create a base population of 8164018; moreover, the U.S. Census Bureau estimates 8113542 adults aged ≥18 years in Georgia. Our final sample size exceeds this requirement, ensuring robust detection of the hypothesized effect.

The recruitment and interview were completed via telephone, with respondents having both landline telephones and those who only use mobile phones. Samples for recruitment are created using list-assisted, random digit dialing. Georgia respondents were randomly selected from each household’s non-institutionalized adult population aged ≥18 years.

### Measures

The BRFSS has a core set of questions asked by all states. States are also encouraged to add items of specific interest^30^. During the 2019 BRFSS, Georgia added a special module containing three items to measure the number of persons with bereavement occurring in 2018 or 2019. Analysis of response rates and use in statistical analyses for the bereavement module is detailed in a prior publication^20^. A sensitivity analysis for all primary endpoints using complete-case data (without imputation) is detailed elsewhere^20^.

*Variables – smoking*

Respondents were defined as smokers if they had smoked at least 100 cigarettes in their entire life. Smokers were further categorized into two groups: every day and some days. Respondents who reported having smoked at least 100 cigarettes in their entire life but do not smoke at all now were defined as former smokers. Respondents who reported they had not smoked at least 100 cigarettes in their entire life were defined as never smokers. The outcome variable, smoking status, was coded as: 1 ‘never smokers’, 2 ‘former smokers’, 3 ‘some days smokers’, and 4 ‘every day smokers’.

*Variables – bereavement*

In the 2019 survey, the Georgia BRFSS added a new module containing three items on the topic of bereavement. Participants were asked: ‘Have you experienced the death of a family member or close friend in the years 2018 or 2019?’. Bereavement was coded as 1 (yes) or 0 (no). This item was derived from the Health and Retirement Survey^17^.

*Variables – self-rated health (SRH)*

In each annual BRFSS, the common core questionnaire queries respondents about SRH. It begins with the statement: ‘Now thinking about your health, which includes stress, depression, and problems with emotions, for how many days during the past 30 days was your health not good?’; the number of days was then allocated to two categories of ≥14 days (coded as 1), and <14 days (coded as 0). This item is easy to administer, is valid and reliable, and showed good construct and criterion validity with respect to the 36-Item Short Form Survey. In the mediation analyses, SRH is coded as ‘1’ if the response was ≥14 days, otherwise as ‘0’.

*Covariates*

Covariates consist of age (18–24, 25–34, 35–44, 45–54, 55–64, ≥65 years), gender (male, female), race (Black/African American only, White only, or all other), residence (metropolitan statistical county, non-metropolitan statistical county), education level (did not graduate high school, graduated high school, attended college/technical school, graduated college/technical school), employment status (employed, unemployed, retired, unable to work, homemaker/student), annual income ($) (<15000, 15000 to <25000, 25000 to <35000, 35000 to <50000, ≥50000), and heavy drinking (yes, no).

### Statistical analysis

Statistical analysis begins with a descriptive analysis of smoking categories and the associated characteristics and health behaviors. With the descriptive analysis, potentially relevant variables leading to the co-occurrence of smoking with bereavement were identified. Pearson’s chi-squared test was performed for statistical significance. All analyses were conducted using imputed datasets.

Among the variables used in this study, the non-response rate ranged from 0.48% to 29.85%. To overcome bias due to differential loss of values, multiple imputation techniques were applied to create imputed datasets^31^. Multiple imputation has three elemental phases: imputation, analysis, and pooling. In the imputation phase, 50 copies of the dataset were created with the missing values replaced with imputed values using multiple imputation by chained equations (MICE). The MICE is a practical approach to impute missing data in multiple variables based on a set of univariate imputation models^32^. We also included many covariates in the imputation model. Imputation involves analysis phase followed by pooling. Analysis for each of the 50 complete datasets used the desired statistical method. Pooling refers the combined results obtained from 50 completed datasets. These results were treated as a single multiple-imputation.

The current study employed Baron and Kenny^33^ approach which was adjusted by Iacobucci et al.^34^ to assess mediation based on imputed data. For simplicity, the Mediation model for smoking, bereavement, and self-rated health, codes each as a binary variable and uses generalized structural equation modeling rather than structural equation modeling for mediation analysis^33-35^.

Since smoking (yes, no) and bereavement are binary variables, we used generalized structural equation modeling instead of structural equation modeling for mediation analysis^35^. Inference (standard errors and p-values) about indirect and total effects was performed using a nonlinear combination^28^. The results are presented as coefficients along with 95% confidence intervals (CIs). Since the dependent variable was an ordinal response variable, ordered logistic regression models were performed to analyze the association between smoking category and bereavement with imputed data. The final model was adjusted for gender, age, race/ethnicity, residence, education level, employment status, annual income, and heavy drinking. Since higher rates of smoking were observed among males, the ordered logistic model was used to analyze bereavement co-occurring among current smokers stratified by gender. The results are presented as odds ratios (ORs) along with 95% confidence intervals (CIs). All statistical analyses were conducted using Stata Version 17^36^.

### Mediation modeling

**Figure 1:**
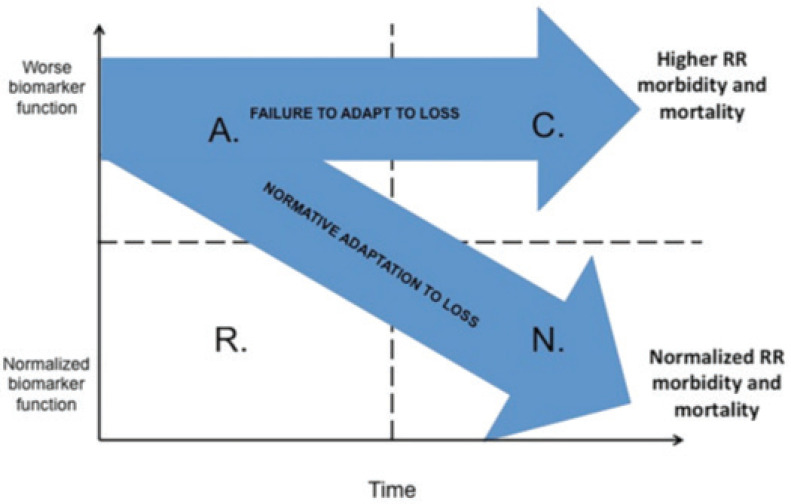
Conceptual framework – model foundation for mediation analysis

Mediation modeling can be applied to measure the influences of both smoking and bereavement on poor SRH using imputed data^37^. Figure 1 illustrates this conceptual framework. In this instance, the concept underlying the Mediation model is derived from evidence describing the physiological adaptation to grief^8^. We hypothesized that smoking is an indirect health behavior bereavement influencing poor SRH. Smoking is a mediating factor for the following reasons: 1) In general, bereavement was associated with negative SRH^19^, and 2) Negative health behaviors were significantly associated with poor SRH^38^.

Hypothesis 3 used biological plausibility as justification for bereavement related injury. Evidence from multiple disciplines such as psychology, neuroscience, immunology, and psychophysiology provide a common mechanism for the symptoms observed among the bereaved. Subsequent morbidity and mortality following the death of a loved one has been associated with one or more of these systems. Figure 1 illustrates two pathways to measure these influences on population-level rates of poor SRH. The concept combines acute and chronic alterations in generic biomarkers. The axes incorporating time (x-axis) with generic biomarker measures (y-axis). In a bereaved population where SRH ranges from poor to excellent. The bifurcating arrow captures the idea that adaptation to loss can be reflected in relative rates (RR) of poor SRH. Its downward course represents a positive adaptation while the unchanged course represents possible persistent or complicated grief. The intersection of SRH, tobacco use, and sociodemographic characteristics add to the probability of continued tobacco use despite a cessation attempt within the context of bereavement.

## RESULTS

Table 1 provides descriptive statistics for the imputed, unweighted analytic sample of 7354 persons aged ≥18 years within the categories of smoking. Smoking at least 100 or more cigarettes in a lifetime was common – 41.4% of the sample. The first row shows the category size and the prevalence of bereavement within each category. While the total proportion of bereaved was 45.6%, the rate of bereavement was greater among all smoker categories: every day (52.2%), some days (53.5%), and former smokers (46.4%). These rates are significantly greater than persons who report never smoking (43.3%). Men are more likely to smoke every day (51.3%) or on some days (52.0%). They have higher rates of smoking in the past (former, 53.03%). As shown in Table 1, the population of never smokers is largely composed of women (62.1%). In each category of smoking, prevalence increased with older age. Social determinants of health such as residence, employment status, education level, and annual income, are shown in the lower half of the table. The distribution of these social determinants is consistent with the state-level population. Within each category of smoking, race, rural residence, unemployment, annual income, and education are distributed differently. While the number of every day smokers is small (n=810), their rates of bereavement were elevated (52.2%).

**Table 1:** Smoking, bereavement and covariates, imputed unweighted data, Behavioral Risk Factor Surveillance System, Georgia, 2019 (N=7354)

*Characteristics*	*Smoking status*				
	*Every day* *(N=810)* *%*	*Some days* *(N=388)* *%*	*Former* *N=1845)* *%*	*Never* *(N=4311)* *%*	*Total* *(N=7354)* *%*
Reporting bereavement**	52.17	53.47	46.36	43.31	45.58
Heavy drinking**					
Yes	14.37	9.27	6.63	2.90	5.43
No	85.63	90.73	93.37	97.10	94.57
Gender**					
Male	51.34	52.02	53.03	37.88	43.91
Female	48.66	47.98	46.97	62.12	56.09
Race**					
Black/African American only	17.95	27.28	15.69	26.07	22.64
White only	71.32	56.43	74.65	58.70	63.97
All other	10.72	16.29	9.67	15.23	13.40
**Age** (years)**					
18–24	3.87	8.99	2.01	9.76	7.13
25–34	13.04	15.75	7.47	12.07	11.22
35–44	16.86	13.43	10.06	13.36	12.92
45–54	18.11	18.43	12.86	15.56	15.31
55–64	27.08	21.35	19.46	17.67	19.35
≥65	21.04	22.05	48.15	31.59	34.08
Residence**					
Metropolitan area statistical county	65.92	68.83	71.92	72.09	71.20
Non-metropolitan statistical county	34.08	31.17	28.08	27.91	28.80
Employment status**					
Employed	45.78	47.35	39.76	49.78	46.70
Unemployed	9.06	7.22	3.69	3.85	4.57
Retired	19.23	18.73	41.58	27.60	29.72
Unable to work	19.48	19.87	10.11	7.81	10.31
Homemaker/student	6.45	6.82	4.87	10.95	8.71
Annual income ($)**					
<15000	22.47	21.05	10.99	11.40	13.03
15000 to <25000	19.01	18.62	26.47	28.16	20.31
25000 to <35000	11.00	11.52	12.86	10.60	11.18
35000 to <50000	12.42	11.99	14.14	12.37	12.80
≥50000	26.35	27.62	44.72	46.23	42.68
Education level**					
Graduated, College/Technical School	14.19	21.18	32.17	39.90	34.14
Attended College/Technical School	28.72	31.80	30.63	24.83	27.08
Graduated High School	35.50	31.77	26.30	24.64	26.63
Did not graduate, High School	21.59	15.25	10.89	10.62	12.14

Pairwise comparison, Pearson’s chi-squared test.

** Significantly different (p<0.01).

Bereavement rates among never smokers were not significantly different between women and men (44.8% vs 40.8%). Table 2 shows details of bereavement rates by gender across separate smoking categories. Among both women and men who smoke every day, there were greater rates of bereavement. Although not statistically significant, female smokers had higher rates of bereavement than males.

**Table 2:** Overall and subgroup estimates of bereavement prevalence per 100, stratified by gender, unweighted data with multiple imputation, Behavioral Risk Factor Surveillance System, Georgia, 2019 (N=7354)

	*Male* *(N=3229)*		*Female* *(N=4125)*	
	*Estimate*	*95% CI*	*Estimate*	*95% CI*
Overall*	43.54	41.49–45.59	47.18	45.43–48.94
Smoking status*				
Never	40.84	38.06–43.63	44.81	42.66–46.95
Former	44.35	40.73–47.98	48.64	44.88–52.39
Some days	52.70	44.39–61.02	54.30	45.83–62.77
Every day	47.80	42.13–53.47	56.77	50.86–62.68

Binary logistic regression.

* Significantly different (p<0.05).

Table 3 presents gender-specific logistic models to compare covariates influencing the likelihood of smoking. Bereaved males were 19% more likely to be smokers. Bereaved females were 32% more likely to be smokers. As anticipated, successively older age was associated with increased odds of smoking for both men and women. This table demonstrates the complexities surrounding smoking, demographic characteristics, and social determinants of health. These results support the decision to pursue a mediation model.

**Table 3:** Ordered logistic regression models – odds of smoking, unweighted data with multiple imputation, Behavioral Risk Factor Surveillance System, Georgia, 2019 (N=7354)

	*Total* *(N=7354)*	*Male* *(N=3229)*	*Female* *(N=4125)*
	*OR (95% CI)*	*OR (95% CI)*	*OR (95% CI)*
**Bereavement**			
No ^®^	1	1	1
Yes	**1.26 (1.13–1.41)**	**1.19 (1.02–1.39)**	**1.32 (1.14–1.54)**
**Age** (years)			
18–24 ^®^	1	1	1
25–34	**3.02 (2.27–4.01)**	**3.06 (2.11–4.45)**	**2.52 (1.61–3.94)**
35–44	**3.77 (2.84–4.99)**	**3.32 (2.27–4.86)**	**3.54 (2.29–5.47)**
45–54	**3.40 (2.58–4.48)**	**2.93 (2.03–4.24)**	**3.30 (2.15–5.08)**
55–64	**3.68 (2.81–4.82)**	**3.03 (2.12–4.35)**	**3.59 (2.35–5.49)**
≥65	**2.96 (2.24–3.92)**	**3.24 (2.22–4.73)**	**2.24 (1.44–3.48)**
**Gender**			
Female ^®^	1	1	1
Male	**1.85 (1.67–2.05)**	-	-
**Race**			
Black/African American only ^®^	1	1	1
White only	**2.12 (1.86–2.41)**	**1.65 (1.36–2.00)**	**2.64 (2.20–3.16)**
All other	1.11 (0.92–1.35)	1.10 (0.84–1.44)	1.04 (0.78–1.38)
**Heavy drinking**			
No ^®^	1	1	1
Yes	**3.81 (3.11–4.67)**	**3.64 (2.72–4.86)**	**3.92 (2.96–5.18)**
**Education level**			
Did not graduate, High School	**2.12 (1.75–2.58)**	**2.53 (1.94–3.29)**	**1.83 (1.38–2.42)**
Graduated High School	**1.97 (1.71–2.26)**	**2.05 (1.68–2.50)**	**1.94 (1.58–2.38)**
Attended College/Technical School	**1.94 (1.70–2.22)**	**2.01 (1.66–2.43)**	**1.90 (1.59–2.28)**
Graduated, College/Technical School ^®^	1	1	1
**Employment status**			
Employed ^®^	1	1	1
Unemployed	**1.68 (1.32–2.15)**	**1.55 (1.10–2.19)**	**1.74 (1.24–2.43)**
Retired	1.11 (0.94–1.31)	1.15 (0.92–1.45)	1.11 (0.88–1.40)
Unable to work	**1.57 (1.31–1.90)**	**1.74 (1.32–2.31)**	**1.46 (1.13–1.88)**
Homemaker/student	0.75 (0.60–0.92)	0.42 (0.24–0.71)	0.83 (0.65–1.06)
**Annual income** ($)			
<15000	**1.74 (1.41–2.15)**	**1.47 (1.08–2.00)**	**2.10 (1.59–2.76)**
15000 to <25000	**1.54 (1.30–1.82)**	**1.40 (1.11–1.77)**	**1.69 (1.34–2.14)**
25000 to <35000	**1.29 (1.08–1.55)**	1.32 (1.00–1.73)	**1.31 (1.02–1.69)**
35000 to <50000	**1.31 (1.10–1.55)**	1.08 (0.85–1.38)	**1.56 (1.22–1.98)**
≥50000 ^®^	1	1	1

Bold indicates statistical significance (p<0.05). ® Reference categories.

**Figure 2:**
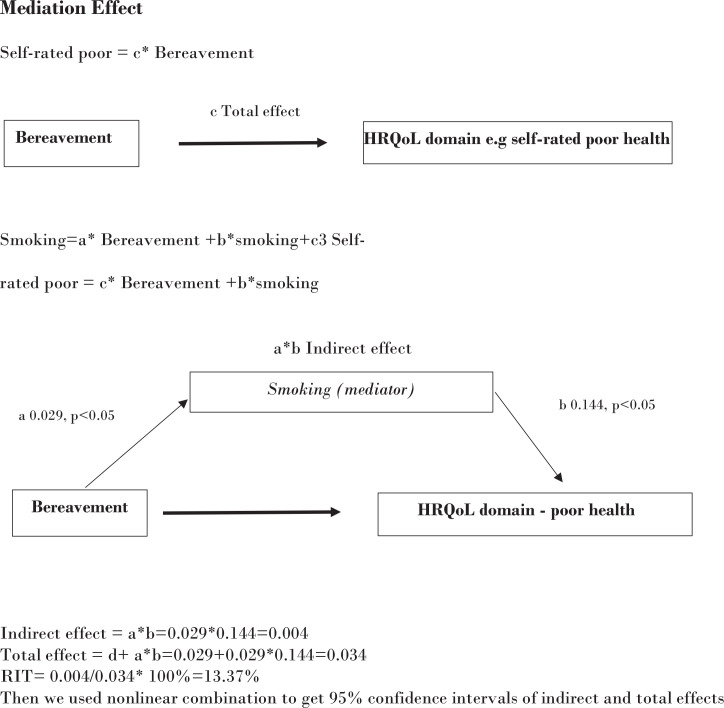
Simple mediation models for bereavement, smoking, and poor self-rated health (Is smoking one possible mechanism underlying the negative health effects of bereavement?)

Figure 2 presents a simple mediation model for bereavement (independent variable), smoking (mediator), and poor SRH (outcome) to quantify direct and indirect effects. In this figure self-rated health is measured by the item: 14 or more days of poor SRH in the prior 30 days. At the top of the figure, a simple model shows bereavement as the only effect on SRH. However, the independent variable (bereavement) can also influence smoking. Smoking then mediates the influence of bereavement on poor SRH. The final product of this model is a ratio of indirect to total effect. Its calculation is contained within the figure. In this analysis, the proportion of smoking (indirect) on bereavement’s total effect is 48.5%. There are insights that come from this model: 1) bereavement plays a direct role on poor SRH; and 2) bereavement has an indirect influence on smoking. Finally, the negative effects of the independent variable on health may be more efficiently addressed with attention to the mediating effects of smoking.

## DISCUSSION

The present study is an analysis of a single year cross-sectional survey from the state of Georgia – the Behavioral Risk Factor Surveillance Survey (BRFSS). The BRFSS is designed to be representative of Georgia’s adult population. With BRFSS, the analysis shows that smokers are significantly more likely to report the death of friends or family in the 24 months prior to survey. Placing a time-bracket around the event of loss is a strategy new to studies of bereavement. The traditional demographic factors such as gender, race, and age were significantly associated with the likelihood of being a current smoker among bereaved persons. Examining a health behavior such as smoking is also new to studies of the bereaved. Smoking and bereavement combine to influence the probability of a report of poor self-rated health (SRH). In the model, smoking is an indirect mediator of poor SRH and combines with bereavement. Combined analyses is a new strategy for defining the context of smoking. These observations bring new perspectives to understanding contextual factors associated with smoking.

### Strengths and limitations

Supplementary file Table S1 lists variables used in this analysis. Missing responses to individual items range from 0% to 29%. Sensitivity analyses of the imputed sample with and without sample weights have been published elsewhere^20^. By combining sampling weights with MI techniques, we create a base population of 8164018. The U.S. Census Bureau estimates that in the state of Georgia there are 8113542 adults aged ≥18 years. Our imputed sample is consistent with census estimates and its standard errors are within National Center for Health Statistics limits, thus ensuring robust detection of hypothesized effects.

A cross-sectional survey of one state in one country has limited generalizability to global populations. The pattern of gender experience with smoking and bereavement is an important starting point for future generalizability studies. In Georgia, 47–49% of adult women report any smoking. In our sample, women who report current smoking also have the highest rates of recent bereavement (54–57%). In this sample, the odds of smoking increase with age among women in parallel with the rate of bereavement^20^. Low-income countries have smaller gender differences in mortality rate when compared to high-income countries. Do gender differences in age-specific mortality rates contribute to the excess smoking rates observed in high-income countries? The Behavioral Risk Factor Surveillance Survey does not measure population health changes in the face of dynamic events such as natural disasters. However, surveillance surveys do consistently measure tobacco use and gender-specific mortality rates. Mortality rates can be an indirect indicator of bereavement in future analyses.

The measurement of bereavement and self-rated health (SRH) is both a limitation and a strength of this analysis. Bereavement was not confirmed. Further research is needed to evaluate the temporal frame used in the BRFSS module. SRH is not a clinical diagnosis. It is widely used in survey research as a shorthand method to gauge wellbeing. However, underneath SRH is an unmeasured but important connection to two forms of grief maladaptation – Prolonged Grief Disorder and Complicated Grief^13-15^. Each of these conditions share symptoms commonly observed with nicotine withdrawal and relapse, i.e. anxiety, depression, insomnia, and difficulty concentrating^7^. The mediation model is a speculative analysis evaluating direct (bereavement) and indirect effects (smoking) on poor SRH. While this model shows that bereavement has the expected robust, negative effect on SRH, smoking appears to exert a mediating role. Longitudinal data are required to fully estimate how smoking influences the association between bereavement and SRH. In BRFSS and other surveillance surveys, smoking is prevalent enough to consider exploring smoking cessation treatment as a strategy to prevent these conditions after a loss.

## CONCLUSIONS

While smoking is a known risk to health, its prevalence among recently bereaved adults is a new observation. Public health surveillance systems need to actively measure this phenomenon to protect both individual and societal health.

## Supplementary Material



## Data Availability

The data supporting this research are available from the Georgia Department of Public Health (https://dph.georgia.gov/phip-datarequest).
